# 2,4-Diamino-Quinazoline, a Wnt Signaling Inhibitor, Suppresses Gastric Cancer Progression and Metastasis

**DOI:** 10.3390/ijms21165901

**Published:** 2020-08-17

**Authors:** Te-Sheng Chang, Chung-Kuang Lu, Yung-Yu Hsieh, Kuo-Liang Wei, Wei-Ming Chen, Sui-Yi Tung, Cheng-Shyong Wu, Michael W. Y. Chan, Ming-Ko Chiang

**Affiliations:** 1 Department of Gastroenterology and Hepatology, Chang Gung Memorial Hospital, Chiayi 61303, Taiwan; cgmh3621@cgmh.org.tw (T.-S.C.); ivan@cgmh.org.tw (Y.-Y.H.); wkliang@cgmh.org.tw (K.-L.W.); 8902030@cgmh.org.tw (W.-M.C.); ma1898@cgmh.org.tw (S.-Y.T.); gi0005@cgmh.org.tw (C.-S.W.); 2 College of Medicine, Chang Gung University, Taoyuan 33302, Taiwan; 3 Department of Biomedical Sciences, National Chung Cheng University, Chiayi 62102, Taiwan; biowyc@ccu.edu.tw

**Keywords:** 2,4-diamino-quinazoline, gastric cancer, Wnt signaling

## Abstract

Gastric cancer (GC) is among the most treatment-refractory epithelial malignancies. Aberrant activation of Wnt/β-catenin-signaling has been implicated in a variety of human cancers, including gastric cancer. Here we report that the elevated expression of lymphoid enhancer binding factor 1 (Lef1) is associated with the TNM (tumor– node–metastasis) stage of gastric cancer. Subsequently, 2,4-diamino-quinazoline (2,4-DAQ), a selective inhibitor of Lef1, was identified to suppress the expression of Wnt/β-catenin target genes such as AXIN2, MYC and LGR5 and result in the suppression of gastric cancer cell growth through the apoptotic pathway. The 2,4-DAQ also exhibited an inhibitory effect on the migration/invasion of gastric cancer cells. Importantly, the treatment of human gastric tumor xenograft with 2,4-DAQ suppressed tumor growth in a nude mouse model. Furthermore, 2,4-DAQ appears effective on patient-derived organoids (PDOs). Transcriptome sequencing analysis also revealed that 2,4-DAQ are more effective on the gastric cancers that exhibit higher expression levels of Wnt-signaling pathway-related genes than their adjacent normal gastric tissues.

## 1. Introduction

Gastric cancer (GC) is one of the most common cancers and the 2nd cause of cancer-related deaths worldwide [1]. In Taiwan, GC was the 7th leading cause of cancer mortality in 2018 [2]. Despite the steady declines in GC incidence rates attributable to higher standards of hygiene, improved food conservation, healthier lifestyle and *Helicobacter pylori* (*H. pylori*) eradication worldwide in the last few decades, almost one million new cases of GC were reported in 2018 [3]. Surgical resection is potentially curative for early stage GCs, but relapse following resection is common. Systemic chemotherapy can improve survival and quality of life for patients with inoperable GCs [4]. However, an effective treatment regimen to substantially reduce the risk of death or progressive disease from GC are not available [5,6].

More than 90% of gastric cancers are adenocarcinoma. It is a multifaceted disease that varies in etiologies, differentiation and molecular pathogenesis [6]. The Lauren classification is the most widely used clinical classification that distinguishes the well-differentiated intestinal-type and the undifferentiated diffuse types [7]. The classification system of the World Health Organization differentiates three major histopathological subtypes of GC as adenocarcinoma, signet ring-cell carcinoma and undifferentiated carcinoma [8]. Recent large-scale studies in molecular subtyping further defined GC into four subtypes, including Epstein–Barr virus-positive tumors, microsatellite unstable tumors, genomically stable tumors and tumors with chromosomal instability across genomic, transcriptomic and proteomic levels [9]. Although these classifications provide a roadmap for patient stratification, they have no impact on the choice of systemic treatment at present.

While the evolutionarily conserved Wnt-signaling pathway plays a crucial role in stem cell maintenance and homeostasis of normal gastric mucosa, aberrant expression of the Wnt-signaling pathway has been implicated in tumorigenesis of GC [10,11]. Our previous study showed that the Wnt-signaling pathway was overexpressed in GC cells, and inhibition of key components of the Wnt-signaling pathway could suppress the proliferation and migration of GC cells [12]. Moreover, the Wnt-signaling pathway is druggable, with a handful of small molecules effective in blocking its functions [13]. T-cell factors/lymphoid enhancer–binding factor (TCF/LEF) family comprising TCF7, TCF7L1, TCF7 L2 and LEF1 are essential cofactors for activation of the Wnt downstream target genes, and the aberrant expression of TCF/LEF family has been implicated in the process of GC tumorigenesis [14]. Through RNA sequencing and TruSeq targeted RNA Wnt-signaling panel analysis, we found that the Lef1 gene was significantly upregulated in GC tissues and the expression level of Lef1 was positively correlated with the cancer stage. The 2,4-DAQ, a potential inhibitor of the β-catenin–TCF/LEF pathway, which can be administered via the peroral route, was shown to inhibit the growth of colorectal cancer cells [15]. In this study, we demonstrate that 2,4-DAQ is effective in inhibiting the growth and invasiveness of GC cells both in vitro and in vivo. Our results suggest that the Wnt-signaling pathway is a druggable therapeutic target in the treatment of GC.

## 2. Results

### 2.1. Aberrant Expression of Lef1 in Gastric Cancer

**Figure 1:**
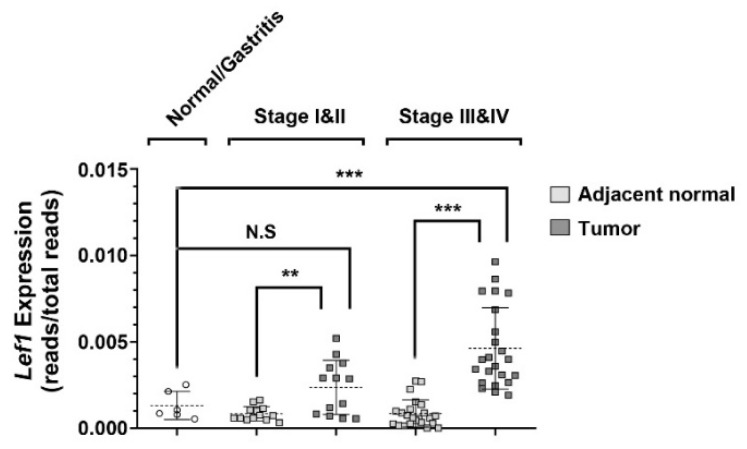
Lymphoid enhancer binding factor 1 (*Lef1*) is overexpressed in gastric cancer. Scatter dot-plot shows the relative *Lef1* expression in 6 normal/gastritis (open circle), 13 early gastric cancer (Stage I & II) and 23 advanced gastric cancer (Stage III & IV) specimens. Data presented as mean with error bars representing SD (** *p* < 0.01, *** *p* < 0.001).

To unravel the pivotal molecular mechanisms promoting the progression of gastric cancer, we collected clinical specimens during the upper GI endoscopic examination with patients’ consents. In addition, we obtained frozen surgically resected gastric cancer tissues from the tissue bank of Chiayi Chang Gung Memorial Hospital. To avoid the interference of the low-quality RNAs that were prepared from the formalin-fixed samples, we utilized the preassembled gene panels (Wnt pathways panels) for targeted RNA sequencing to analyze the expression levels of Wnt signaling-related genes in GC. This methodology enables the high-throughput analysis of multiple genes on low-quality RNA samples. The study cohort was divided into early stage (Stage I and II) and late-stage (Stage III and IV) groups. From statistical analysis, we identified Lef1, a transcription factor in the Wnt-signaling pathway, whose expression level in most the cancerous lesions was well above the level found in the adjacent normal tissues, as well as the level found in the gastric tissues of other non-cancer patients (Figure 1). This result indicated that Lef1 became significantly upregulated during the progression of gastric cancer and prompted us to search for the Lef1 inhibitors that may suppress the progression of gastric cancer.

### 2.2. Inhibitory Effects of Wnt Signaling Inhibitors on Gastric Cancer Cells

**Figure 2:**
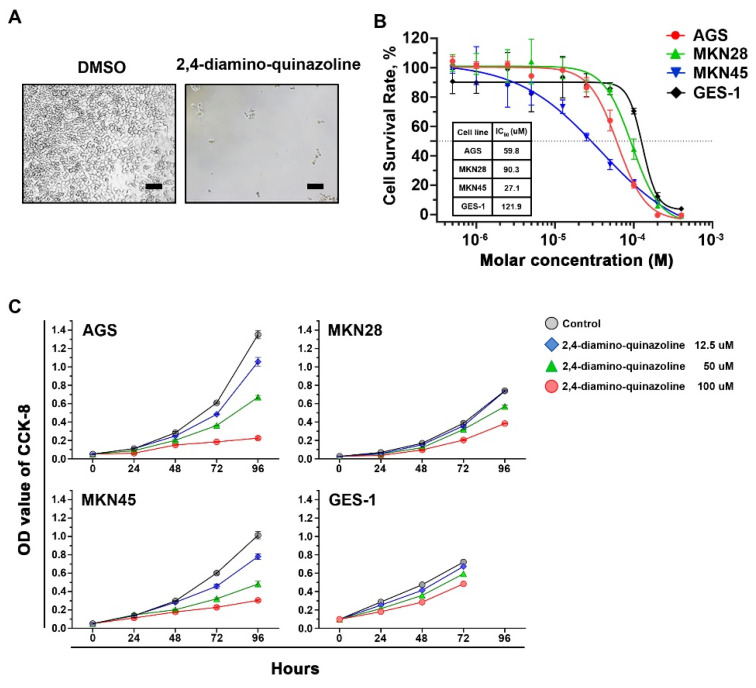
Effects of the β-catenin-T-cell factors/lymphoid enhancer–binding factor (TCF/LEF) pathway inhibitors on cell viability of gastric cancer cells (AGS). (**A**) Representative morphology of AGS cells cultured for 96 h in the presence of 2,4-DAQ. Scale bar: 100 μm; (**B**) calculated IC50 growth inhibition values of 2,4-DAQ in three gastric cancer cell lines and immortalized human epithelial cells (GES-1); (**C**) dose- and time-dependent inhibition effect of 2,4-DAQ on three gastric cancer cell lines and GES-1 was evaluated by CCK-8 assay.

Currently, many Wnt-signaling inhibitors have been tested in clinical trials on various cancers. However, there were no reports on their effects for treating gastric cancer. We tested some available compounds (Table S1) that inhibit Wnt signaling on established gastric cancer cell lines and an immortalized gastric cell line (GES-1). They all suppress the growth of gastric cancer cell lines. However, only 2,4-DAQ, an inhibitor of the β-catenin-TCF/LEF pathway, exhibited a more substantial inhibitory effect toward the gastric cancer cell lines than the immortalized gastric cell line. We first examined the effects of 2,4-DAQ on the growth of gastric cancer cell lines. Cell morphology was captured for each treatment (100 μM) via brightfield microscopy (Figure 2A), and the IC50 (the concentration that inhibits the survival of cells by 50%) values were calculated following incubation with various concentrations of 2,4-DAQ. The 2,4-DAQ showed dose-dependent growth inhibition effects on gastric cancer cell lines (AGS and MKN45) at low micromolar concentrations (Figure 2B).

**Figure 3:**
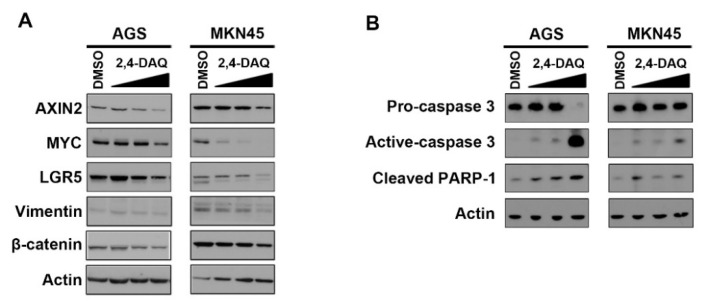
2,4-DAQ regulates Wnt/β-catenin responsive genes and induces caspase 3-dependent apoptosis in gastric cancer cells. The AGS and MKN45 cells were treated with different concentrations of 2,4-DAQ (100, 200 and 300 μM) or control (DMSO) for 48 h. Total lysates of cells were analyzed by western blot analysis with specific antibodies against (**A**) Wnt/β-catenin pathway (**A**,**B**) apoptosis-related proteins as indicated. Actin represents the loading controls.

The growth curves indicated that AGS and MKN45 cells were sensitive to 2,4-DAQ, and the growth inhibition was in a dose-dependent and time-dependent manner. On the other hand, the IC50 of 2,4-DAQ on GES-1 cells was higher, indicating that GES-1 was more resistant to 2,4-DAQ than AGS and MKN45 cells (Figure 2B,C). To further confirm the inhibitory effects of 2,4-DAQ on the Wnt/β-catenin pathway, we assessed the effects of 2,4-DAQ treatment on the expression of Wnt/β-catenin downstream target genes. The expression level of Wnt/β-catenin downstream pathway genes, including AXIN-2, MYC, vimentin and LGR5, was examined in AGS cells at the protein level, which decreased in response to 2,4-DAQ treatment in a dose-dependent manner (Figure 3A). Additionally, 2,4-DAQ downregulated the expression of two other mesenchymal markers, N-cadherin and Snail (Figure S1). We also assessed the expression of several apoptosis-related proteins in AGS and MKN45 cells treated with different concentrations (100–300 μM) of 2,4-DAQ for 48 h. Apoptosis was brought about in a dose-dependent manner, as indicated by the presence of cleaved caspase-3 and cleaved PARP in these cell lines (Figure 3B). These results showed that 2,4-DAQ inhibited cell growth and induced apoptosis of the human gastric cancer cell lines.

### 2.3. Effect of 2,4-DAQ on Colony Formation, Cell Migration and Invasion of Gastric Cancer Cells

**Figure 4:**
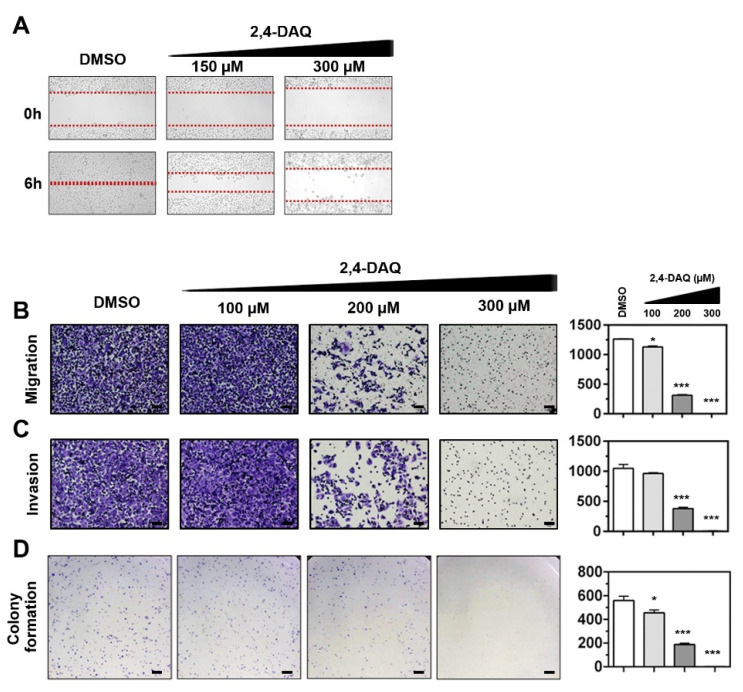
2,4-DAQ modulates the colony formation, migration and invasion abilities of AGS cells. The gastric cancer cells were treated with different concentrations of 2,4-DAQ or control (DMSO) and (**A**) processed for wound-healing assay. (**B**) Transwell migration assay (without Matrigel); (**C**) invasion assay (with Matrigel); (**D**) colony formation assay. Data presented as mean with error bars representing SD (* *p* < 0.05; *** *p* < 0.001). Scale bar: 100 μm.

To investigate the antimigratory effects of 2,4-DAQ, we subjected 2,4-DAQ-treated-AGS cells to wound healing assay with standard culture inserts. The vehicle (DMSO)-treated AGS cells were observed to migrate towards the empty area after 6 h of incubation. On the other hand, the 2,4-DAQ-treated cells showed a significant dose-dependent reduction in their migration ability (Figure 4A). Similarly, the Transwell migration assay showed that the number of migrating cells in the 2,4-DAQ-treated group was significantly lower than that in the vehicle control, suggesting that 2,4-DAQ reduced the AGS cell migration (Figure 4B). Next, we investigated the effect of 2,4-DAQ on the cell invasive properties using Matrigel-coated Transwell. As shown in Figure 4C, the number of cells crossed Matrigel during the 2,4-DAQ treatment was significantly decreased compared to the number of cells in the control group. These results revealed that 2,4-DAQ exhibited a significant inhibitory effect on the migration and invasion of AGS cells. In addition, as shown in Figure 4D, in the colony formation assay, much fewer colonies were observed in 2,4-DAQ-treated cells than in control cells.

### 2.4. 2,4-DAQ Inhibits Xenograft Tumor Growth In Vivo

**Figure 5:**
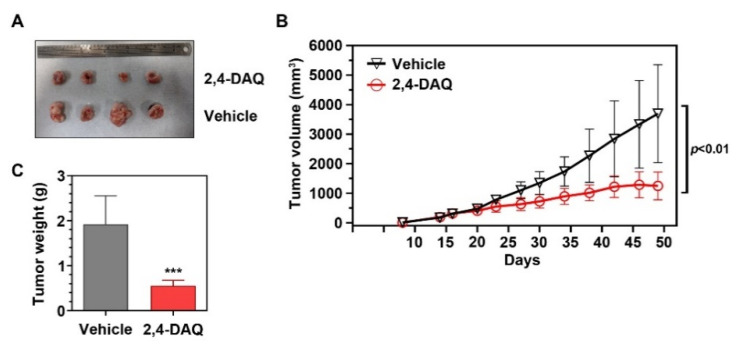
Effects of 2,4-DAQ on tumor volume and tumor weight in gastric cancer cells (MKN45) xenograft nude mice. (**A**) Representative images showing injected xenograft tumors in nude mice at Day 49 post subcutaneous injection. (**A**,**B**) tumor growth and (**C**) tumor weight evaluation of 2,4-DAQ vs. vehicle group. Symbol *** indicates *p* < 0.001.

To determine whether Wnt-signaling inhibitors have inhibitory effects on gastric tumor growth in vivo, we administered 2,4-DAQ to nude mice with MKN45 xenografts. Fifty milligrams per kilogram 2,4-DAQ or vehicle (DMSO) was injected intraperitoneally (i.p.) into nude mice bearing 400 mm^3^ tumors every three days for 12 days. The 2,4-DAQ treatment did not show any effect on the survival rate and the bodyweight change of the animals (Figure S2). However, 2,4-DAQ treatment resulted in a significant reduction in both tumor volume (Figure 5A,B) and tumor weight (Figure 5C) compared to the tumors of the vehicle-treated group. These data suggest that 2,4-DAQ inhibits tumor growth in vivo. Taken together, our in vitro and in vivo results indicate that 2,4-DAQ has the potential to inhibit tumorigenesis.

### 2.5. Inhibitory Effects of 2,4-DAQ on Patient-Derived Organoids

**Figure 6:**
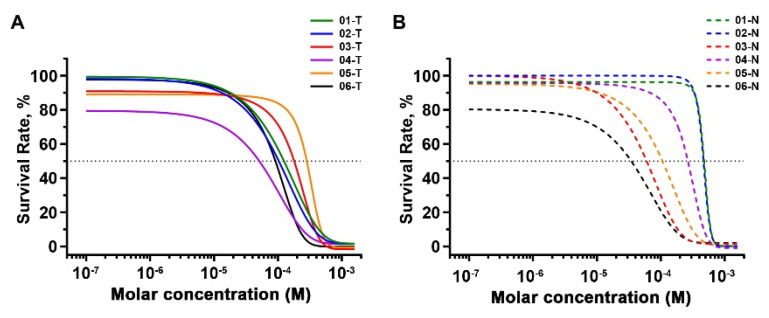
Effects of the 2,4-DAQ on cell viability of gastric cancer (GC) organoids. Organoids viability assay was performed to determine the growth inhibition values of 2,4-DAQ. (**A**) Dose–response of tumor-derived organoids and (**B**) nonneoplastic lesions-derived organoids proliferation to 2,4-DAQ treatment measured at 6 days. All data presented as mean ± SD, *n* = 3.

Currently, many Wnt-signaling inhibitors have been tested in clinical trials on various cancers, yet there was no report on their effects on gastric cancer. To further explore the possibility of applying Wnt-signaling inhibitors to treat gastric cancer, we treated organoids derived from six gastric cancer patients (Figure S3 and Table S2) with 2,4-DAQ at different concentrations for 120–144 h and analyzed cell viability through alamarBlue assay (Figure 6A). As a comparison, organoids generated from nonneoplastic lesions of the same patients were treated with the same drugs (Figure 6B). The IC50 (the concentration that inhibits the survival of cells by 50%) values were calculated following incubation with various concentrations of the compound (Table 1) and appeared to vary in organoids from different patients.

**Figure 7:**
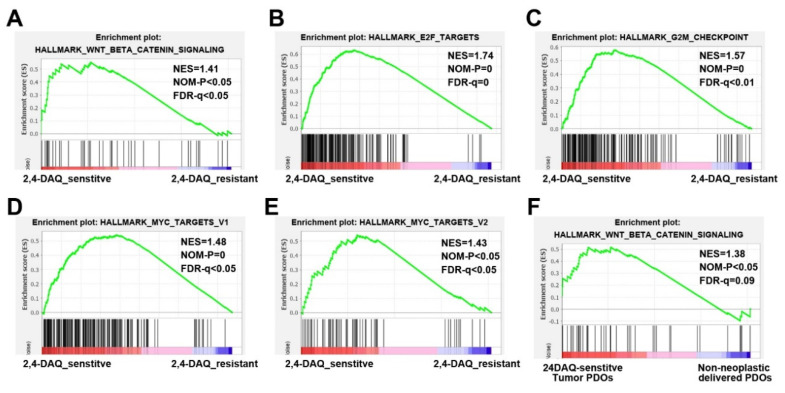
Gene set enrichment analysis (GSEA) of transcriptional profiles in patient-derived organoids. GSEA analysis using hallmark gene sets from the molecular signature database for the transcriptional difference in patient-derived organoids. (**A**) Enrichment plots of genes in hallmark_Wnt_beta-catenin-signaling; (**B**) hallmark_E2F_targets; (**C**) hallmark_G2M_checkpoint; (**D**) hallmark_MYC targets_V1 and (**E**) hallmark_MYC targets_V2 gene sets showed significant enrichment in 2,4-DAQ-sensitive tumor-derived organoids (ID: 01, 02, 04) versus 2,4-DAQ-resistant tumor-derived organoids (ID: 03, 05, 06). Enrichment plot of genes in (**F**) hallmark_Wnt_beta-catenin-signaling gene sets showed significant enrichment in 2,4-DAQ-sensitive tumor-derived organoids versus 2,4-DAQ-sensitive nonneoplastic lesions-derived organoids. NES—normalized enrichment score; NOM-P—nominal *p*-value; FDR-*q*—false discovery rate *q*-value.

Based on whether the 2,4-DAQ’s IC50 in the gastric cancer organoids were higher or lower than that in the organoids derived from the adjacent normal tissues of the same patients, we distinguished these tumor organoids into 2,4-DAQ-sensitive (ID: 01, 02, 04) and 2,4-DAQ-resistant tumor organoids (ID: 03, 05, 06). To identify molecular features in gene expression patterns that were associated with organoids’ response to 2,4-DAQ, we performed transcriptome sequencing on the tumor-derived and the nonneoplastic lesions-organoids from these 6 patients that passed quality control. The transcriptomic profile was then further analyzed with a gene set enrichment analysis (GSEA). This analysis used the hallmark gene sets that cover well-defined biologic states and processes in the Molecular Signature Database (Broad Institute). Interestingly, the analysis revealed significant enrichment of gene sets associated with hallmark_Wnt beta-catenin-signaling in 2,4-DAQ-sensitive tumor organoids versus 2,4-DAQ-resistant tumor organoids (Figure 7A). Gene sets representative of hallmark_E2F_targets, hallmark_G2M_checkpoint, hallmark_MYC targets_V1 and hallmark_MYC targets_V2 were also enriched in the same organoids (Figure 7B–E). It indicated that when compared to 2,4-DAQ-resistant tumor organoids, 2,4-DAQ-sensitive tumor organoids expressed genes in the Wnt-signaling pathway at a significantly higher level, as well as many proliferation-related genes, including E2F-target genes, G2M-checkpoint genes and c-Myc target genes. Furthermore, we also found enrichment of hallmark_Wnt_bata-catenin-signaling in 2,4-DAQ-sensitive tumor organoids compared to the organoids derived from the nonneoplastic lesions of the same patients (Figure 7F). Collectively, our data implied that activation of the Wnt/β-catenin pathway contributed to 2,4-DAQ-sensitivity.

## 3. Discussion

Like most cancers, gastric cancer is difficult to cure unless it is found at an early stage when resection is feasible. Surgery is the mainstay of curative treatment for gastric cancer. However, tumor recurrence and relapse are likely to occur due to undetectable residual cancer cells. Various chemotherapeutic drugs, including 5-FU (fluorouracil), cisplatin, oxaliplatin, capecitabine and paclitaxel, are commonly used in gastric cancer treatment when curative resection cannot be achieved. Although there is evidence that either adjuvant or palliative chemotherapy is beneficial for gastric cancer patients, the overall prognosis remains dismal as gastric cancer has not been particularly sensitive to any of these chemotherapy regimens. Although the US Food and Drug Administration (FDA) and the European regulatory authorities have approved trastuzumab (Herceptin), a target therapy drug, in combination with chemotherapy for treating metastatic gastric cancer, the improvement of median survival was marginal [16]. As a result, efforts to uncover the molecular pathways involved in gastric carcinogenesis are crucial for optimizing the therapy efficiency of this intractable cancer.

It was postulated that the dysregulation of surrounding signaling molecules stimulates the cancer stem cell proliferation and contributes to tumorigenesis [17]. In addition, Wnt signaling has been demonstrated to be crucial for the maintenance of normal stem cells during the development of the gastrointestinal tract and nervous system. Targeting the Wnt-signaling components appears to be a promising approach [18]. Currently, many Wnt-signaling inhibitors were tested in clinical trials on various cancers, yet there was no report on their effects on gastric cancer.

Studies have shown that Quinazoline and its derivatives, which belong to organic heterocyclic compounds, possess a broad range of biopharmaceutical activities, including anti-inflammation [19], antimicrobial [20,21], antimetabolic syndrome [22,23,24], anti-convulsant [25,26] and anti-psychotics activity [27]. In recent years, quinazoline derivatives have been developed as drugs against different tumors, including FDA-approved erlotinib, gefitinib, vandetanib and lapatinib [28,29,30,31,32]. Researchers have further extensively used quinazoline derivatives to develop inhibitors that can act on tyrosine kinases, serine–threonine kinases, p53 regulators, the folic acid pathway and signaling pathways to reduce cancer cell growth [33]. Wyeth reported a structurally related series of quinazolines as potent inhibitors targeting β-catenin-TCF/LEF-mediated transcription and proliferation in colorectal cancer cell lines and xenograft models [15,34]. Our study has found that the elevated expression of LEF1—a key component of the Wnt-signaling pathway—correlates with the occurrence of gastric cancer. The results highlight the possibility of using quinazoline derivatives as β-catenin-TCF/LEF-signaling inhibitors to treat gastric cancer patients.

In this study, our results suggested that 2,4-DAQ inhibited the viability and clonogenicity of gastric cancer cell lines (AGS and MKN45) in a dose-dependent manner and promoted the apoptosis of AGS and MKN45 cells, which can be verified by the presence of apoptotic markers (caspase activation and cleavage of PARP-1). Moreover, 2,4-DAQ decreased the expression of critical β-catenin target genes, including MYC and LGR5. Interestingly, LGR5 was previously implicated as a CSC marker in GC [35,36] and MYC promotes cell proliferation [37]. Therefore, these results support that 2,4-DAQ exerts its inhibitory effects on cancer cell stemness and viability through suppressing the canonical Wnt signaling.

It is well-established that the Wnt/β-catenin pathway plays a role in tumor cell epithelial-mesenchymal transition (EMT), which could promote cancer metastasis [38,39]. The results of wound-healing assay and Transwell migration/invasion experiments both indicated that 2,4-DAQ reduced the migration and invasion activity of GC cells. It should be noted that the effect of 2,4-DAQ on cell growth could also affect the outcome of the migration and invasion assay. However, since 2,4-DAQ could decrease the expression of epithelial–mesenchymal transition markers (vimentin, N-cadherin and snail) in a dose-dependent manner, suggesting that 2,4-DAQ directly attenuated the metastatic characteristics of GC cells. Importantly, our results demonstrate the potential of using 2,4-DAQ as a therapeutic agent against gastric cancer in an in vivo xenograft model using human GC cell lines. The 2,4-DAQ treatment significantly reduced the tumor growth rate and final tumor weight in xenograft. Moreover, no significant changes in activity, mortality and bodyweight of the mice were observed, indicating that 2,4-DAQ -associated toxicity was minimal. However, further evaluation of the therapeutic safety of 2,4-DAQ is still required.

The high fatality of GC due to the lack of effective drugs raises the urgent need for good in vitro models that encompass unique subtypes for precision medicine development. The traditional two-dimensional in vitro culture system lacks physiologic simulations and as a result, it is poor in modeling human development and disease. Although many animal models display similar clinical symptoms to that of human patients, the experimental complexity and inability to study the aspects of human development and disease have restricted their widespread use. The recent development of three-dimensional organoid culture has integrated a more comprehensive mechanobiological perspective into the studies of organ development and carcinogenesis [40,41]. The organoid is a multicellular system that recapitulates the architecture and functionality of native tissue [42]. This novel technique has allowed researchers to develop different models of human cancer, including GC. Moreover, an organoid may be used to accurately predict the patients’ responses to therapeutic drugs before the treatment [43,44].

In this study, we successfully established GC organoids and nonneoplastic gastric organoids from surgically resected tissue of patients with gastric adenocarcinoma and its adjacent gastric epithelium. Next, we set out to analyze the response of these organoid lines to 2,4-DAQ treatment. Base on the results of cell viability experiments, we distinguished tumor organoids into 2,4-DAQ-sensitive and 2,4-DAQ-resistant tumor organoids. We reasoned that the patients in the 2,4-DAQ-sensitive group would respond to 2,4-DAQ treatment better since their normal gastric epithelium could tolerate higher concentrations of 2,4-DAQ than the GC. Interestingly, the GSEA analysis of these patient-derived organoids revealed that genes related to hallmark_Wnt_beta-catenin-signaling and cell proliferation such as hallmark_E2F_targets, hallmark_G2M_checkpoint, hallmark_MYC targets_V1, and hallmark_MYC targets_V2 were highly enriched in the 2,4-DAQ-sensitive tumor organoids versus the 2,4-DAQ-resistant tumor organoids. Moreover, we found that genes of the hallmark_Wnt_beta-catenin-signaling were significantly enriched in the 2,4-DAQ-sensitive tumor organoids versus their paired nonneoplastic organoids. These data indicated that the 2,4-DAQ-sensitive tumor organoids were derived from the tumors which were impacted by dysregulation of Wnt signaling.

In the past decade, molecular profiling studies have enabled us to understand better about the complexity and the heterogeneity of GC; however, up to now, few target-directed options have been approved for treating GC. Part of the reasons being that an appropriate molecular selection of patients has not been conducted in many target driven clinical trials. Our study has shown that, with the preselection of suitable patients using PDOs for gene profiling and pre-clinical drug testing, many signaling-targeting drugs, including 2,4-DAQ, may succeed in demonstrating a clinical benefit in GC.

In conclusion, our results indicate that 2,4-DAQ exerts its potent, selective antitumor effect primarily through inhibition of the Wnt/β-catenin-signaling pathway on GC cells, suggesting the potential therapeutic usage of 2,4-DAQ in clinical applications.

## 4. Materials and Methods

### 4.1. Cell Lines and Gastric Cancer Tissue

All the gastric cancer cell lines and human gastric epithelial cell line (GES-1) used in this work were cultured in RPMI-1640 (Thermo Fisher Scientific, Waltham, MA, USA), supplemented with 10% fetal bovine serum, 100 units/mL penicillin and 100-mg/mL streptomycin (Thermo Fisher Scientific), in a 5% CO_2_ atmosphere at 37 °C. The L-WRN cell was purchased from American Type Culture Collection (ATCC^®^, CRL 3276™) and cultured in DMEM supplemented with 10% fetal bovine serum (FBS) and 1% P/S, 0.5-mg/mL hygromycin B (Roche, Basesl, Switzerland) and 0.5-mg/mL G-418 and maintained in an incubator with a humidified atmosphere of 5% CO_2_ at 37 °C.

Formalin-fixed tissue samples for Targeted sequencing and Transcriptome sequencing were collected from 67 patients whose mean age was 68.1 ± 10.0 years between 2010 and 2014 at Chang Gung Memorial Hospital, Chiayi, Taiwan. Stomach tissues for organoid cultures were obtained from patients who were diagnosed with gastric cancer and had received a curative gastrectomy between 2018 and 2019 at Chang Gung Memorial Hospital with informed consent. The acquisition and use of clinical specimens in this study were approved by the Institutional Review Board of Chang Gung Medical Foundation (IRB approval number: 102–1996B, approval date: 16 July 2013; 201700171A3, approval date: 23 March 2013).

### 4.2. Targeted Sequencing and Transcriptome Sequencing

Total RNA was isolated from stomach tissue using the TRIzolTM reagent (Thermo Fisher Scientific, Waltham, MA, USA) according to the manufacturer’s instructions. The concentration and integrity numbers of purified RNA samples were assessed by Qubit 2.0 fluorometer (Thermo Fisher Scientific) and 2200 TapeStatio (Agilent Technologies, Santa Clara, CA, USA), respectively. The targeted sequencing and transcriptome sequencing were performed following the protocol reported in previous studies [45]. Briefly, Sequencing-ready libraries of amplified targeted genes were prepared using TruSeq targeted RNA Wnt pathway panel kit (Illumina, San Diego, CA, USA) by following the manufacturer’s manuals [46]. Sequencing was carried in a MiSeq sequencer (Illumina), and a DESeq package was used to remap the sequencing reads to the human genome (hg19). The expression level of each gene was represented by gene read number/total specimen read number. The libraries for transcriptome sequencing analysis were prepared using Agilent SureSelect strand-specific mRNA library preparation kit following the manufacturer’s directions and were sequenced in 100-bp paired-end reads on Illumina MiSeq. Mapping, annotation and calculation of gene expression level (fragments per kilobase of transcript per million mapped) were performed using CLC Genomic Workbench v. 8.5 (Qiagen, Hilden, Germany).

### 4.3. Cell Proliferation Assay (Cell Counting Kit-8)

Cell viability was evaluated by a cell counting Kit-8 (CCK-8) (Sigma-Aldrich, St. Louis, MO, USA) assay. The cells of test groups were plated at a density of 1 × 103 cells/well in 96-well culture plates and were analyzed at different time points. A volume of 10 μL/well of CCK-8 solution was added into the plate. After incubation at 37 °C for 1 h, the absorbance was measured at 450-nm using a microplate reader. Each assay was completed in triplicate wells, and each experiment was repeated three times.

### 4.4. Cell Proliferation Assay (alamarBlue)

Cell viability was evaluated by alamarBlue (Bio-Rad Laboratories, Hercules, CA, USA) assay. The organoids of test groups were plated in 96-well culture plates and were analyzed at different time points. A volume of 200 μL/well of 10% alamarBlue solution was added into the plate. After incubation at 37 °C for 2–5 h, the fluorescence was detected using a microplate reader (using an excitation between 530–560 and an emission at 590 nm). Each assay was completed in triplicate wells, and each experiment was repeated three times.

### 4.5. Western Blot Analysis

Cell lysates were prepared by incubating cells with lysis buffer (10-mM sodium phosphate, pH 7.2, 100-mM NaCl, 2-mM EDTA, pH 8.0, 1% NP-40 and protease inhibitor cocktail) for 15 min on ice and clearing by centrifugation for 15 min at 4 °C. The western blot analysis of each protein was performed as described previously. Levels of proteins were normalized against levels of actin proteins and represented as relative fold change over levels in control samples.

### 4.6. Colony Formation Assay

Cells treated with 2,4-DAQ after 48 h were seeded in 6-well plates at a density of 2000 cells/well and incubated for four days. The colonies were fixed with methanol at room temperature for 10 min, stained with 0.4% crystal violet for 10 min, and finally, positive colony formation (more than 50 cells/colony) was counted, and the colony formation rate was calculated.

### 4.7. Wound-Healing Assay

For the wound-healing assay, AGS cells were treated with 2,4-DAQ for 24 h. Then the cells were washed twice with PBS and seeded into each side of the Culture-inserts 2 Well (ibidi, Gräfelfing, Germany) at a density of 1 × 10^5^ cells/mL. After 24 h of incubation in regular medium, culture inserts were carefully removed. Photographs were taken at 0, 6 h to observe the wound healing phenomenon.

### 4.8. Cell Migration and Invasion Assay

For the Transwell-migration assay, AGS cells were treated with2,4-DAQ (0 to 300 μM) for 24 h then were serum-starved for 6 h. Then, 5 × 10^4^ cells were seeded onto the upper chamber (8-μm pore size) of Millicell^®^ tissue culture plate well inserts (Merk Millipore, Billerica, MA, USA) without Matrigel and incubated for 24 h with medium containing 10% FBS in the bottom of the chamber. Residual cells on the upper side of chambers were removed by scraping with cotton swabs and the cells that attached to the lower side of the membrane were fixed with methanol for 10 min and stained with 0.4% crystal violet/50% methanol for 10 min. The invaded cells were counted in four random fields (×100) per filter, and photographs were taken by microscopy. For the invasion assay, the Matrigel-coated chambers purchased from BD Biosciences were used. Protocols were similar as described in the Transwell-migration assay.

### 4.9. Xenograft Studies

Athymic nude mice (8-week, male) were purchased from the National Laboratory Animal Center (Tainan, Taiwan). MKN45 cells (2 × 10^6^/mouse) were mixed with an equal volume of Matrigel (Becton Dickinson) and injected subcutaneously into the posterior leg of athymic nude mice. The tumor size was measured twice per week in two dimensions with calipers and calculated using the formula (length × width^2^)/2. Once the tumor size reached around 400 mm^3^, which occurred 3 weeks after cell injection, the mice were randomly assigned into 2,4-DAQ treatment groups with a dose of 50 mg/kg and a vehicle control group. The 2,4-DAQ in 500-μL saline (with 2.5% DMSO) was injected intraperitoneally into each mouse every three days for 12 days. Control mice were intraperitoneally injected with 500-μL saline (with 2.5% DMSO) as a vehicle control every three days for 12 days. Tumors were harvested 49 days after inoculation and individually weighed. All of the animal experiments were performed with the approval of the Institutional Animal Care and Use Committee at our Laboratory Animal Center, Department of Medical Research, Chang Gung Memorial Hospital at Chiayi (IACUC no: 2017011301, approval date: 17 April 2017).

### 4.10. Generating Wnt3a and Wnt3a/R-Spondin 3/noggin Conditioned Medium

Wnt3a/R-spondin 3/noggin-conditioned induction medium was generated using L-WRN cells, respectively, according to the manufacturer’s protocol [47,48]. The two conditioned media were filtered using 0.22-μm filters and stored at 4 °C until usage.

### 4.11. Organoid Culture

Fresh tumor tissue and adjacent normal samples were processed as previously described [49]. The tumor tissue was cut into <5 mm^2^ stripes, washed with cold Hank’s buffered saline solution (HBSS) at least ten times and subsequently digested with Liberase (TM grade; Roche, Basesl, Switzerland) for 30 min at 37 °C with vigorous pipetting every 5 min. The supernatant was collected and centrifuged at 200× *g* for 3 min at 4 °C. The cell pellet was suspended with Matrigel (growth factor reduced; BD Biosciences) and dispensed into 24-well culture plates (30 μL Matrigel/well) and overlaid with organoid culture medium: advanced DMEM/F12 with 50% Wnt-3A-conditioned medium, HEPES (Thermo Fisher Scientific), GlutaMAX (Thermo Fisher Scientific), penicillin/streptomycin (Thermo Fisher Scientific), B27 supplement (Thermo Fisher Scientific), N2 supplement (Thermo Fisher Scientific), n-acetylcysteine (Sigma-Aldrich), nicotinamide (Sigma-Aldrich), recombinant human EGF (Peprotech, Cranbury, NJ, USA), recombinant human R-spondin1 (Peprotech), recombinant mouse noggin (Peprotech), recombinant human FGF10 (Peprotech), gastrin (Sigma-Aldrich), transferrin (Sigma-Aldrich), insulin (Sigma-Aldrich), A83-01(ALK5 inhibitor, Sigma-Aldrich), SB 202190 (p38 MAPK inhibitor, Sigma-Aldrich), chir 99021 (GSK-3 inhibitor, Sigma-Aldrich) and Y-27632 dihydrochloride (rho kinase inhibitor, Sigma-Aldrich). In some experiments, 50% L-WRN-conditioned medium replaced 50% Wnt-3A-conditioned medium, R-spondin1 and noggin. The organoid culture medium was refreshed every three to seven days.

Adjacent normal samples from gastric cancer patients were washed with cold HBSS and fat and connective tissue were removed. Samples were cut into small pieces (approximately <5 mm^3^, washed with cold HBSS until the supernatant was clear and incubated with the chelating solution (5.6-mM Na_2_HPO_4_, 8.0-mM KH_2_PO_4_, 96.2-mM NaCl, 1.6-mM KCl, 43.4-mM sucrose, 54.9-mM D-sorbitol, 0.5-mM DL-dithiothreitol and 2-mM EDTA) for 30 min at 4 °C on a shaking platform. After shaking, tissue pieces could settle for 1–2 min, and the supernatant was then removed. The tissue pieces were carefully transferred in the middle of a sterile petri dish and pressure was applied until the area around the tissue pieces appeared cloudy. Isolated glands were resuspended in cold advanced DMEM/F12, collected in a tube and allowed to settle for 1–2 min by gravity before the supernatant containing most of the isolated glands was transferred to a new tube. The glands were counted under a microscope, washed twice with cold Advanced DMEM/F12 and centrifuged at 200× *g* for 3 min at 4 °C. The pellet was suspended with Matrigel and dispensed into 24-well culture plates (30 μL Matrigel/well) and overlaid with the organoid culture medium as described earlier.

### 4.12. Gene Set Enrichment Analysis (GSEA)

GSEA was performed using the GSEA software (v4.0.3, Broad Institute, Cambridge, MA, USA) on the normalized gene expression data (FPKMs) from transcriptome sequencing following the protocol from the Broad Institute Gene Set Enrichment Analysis website. Briefly, the analysis in our study was performed as two separate comparisons: (1) 2,4-DAQ-sensitive organoids compared to 2,4-DAQ-resistant organoids and (2) 2,4-DAQ-sensitive organoids compared to nonneoplastic lesions delivered organoids. The gene sets were adopted from the Molecular Signatures Database (MSigDB, v7.0, Broad Institute, Cambridge, MA, USA) within the Hallmarks 50 gene sets. The GSEA software was run with 1000 permutations for statistical significance estimation and the signal-to-noise metric between the two phenotypes for ranking all genes, and other parameters were set to default values. The GSEA software calculated the enrichment score (ES) reflecting the degree to which the gene set is over-represented in our gene expression data. Normalized enrichment score (NES) is the ES normalized for the sizes of the gene set. Significantly enriched gene sets were considered with nominal (NOM) *p*-value < 0.05 and false discovery rate (FDR) *q*-value < 0.05.

### 4.13. Statistical Analysis

All experiments were repeated at least three times. Results are presented as the mean± SD and were analyzed using a Student’s t-test. To examine the clinical correlation of the above findings, we performed a linear regression to examine the correlation between the expression of those genes. Univariate and multivariate analyses were also performed to examine the expression of those genes with clinical parameters.

## Figures and Tables

**Table 1:** IC_50_ values for 2,4-diamino-quinazoline for GC PDOs.

Patient ID	IC_50_ (μM)	
	Tumor	Non-Neoplastic
01	110.0	464.5
02	82.5	456.8
03	142.5	54.3
04	24.1	242.0
05	247.6	91.8
06	81.9	17.3

## Supplementary Materials

Supplementary materials can be found at https://www.mdpi.com/1422-0067/21/16/5901/s1.
