# Supplementary material for: 2,4-Diamino-Quinazoline, a Wnt Signaling Inhibitor, Suppresses Gastric Cancer Progression and Metastasis
Source: Int J Mol Sci. 2020 Aug 17;21(16):5901. doi: 10.3390/ijms21165901 (PMC7460562; doi:10.3390/ijms21165901)
Supplement: Supplementary file 1 [file ijms-21-05901-s001.pdf]

Supplementary Information

Supplementary Figures

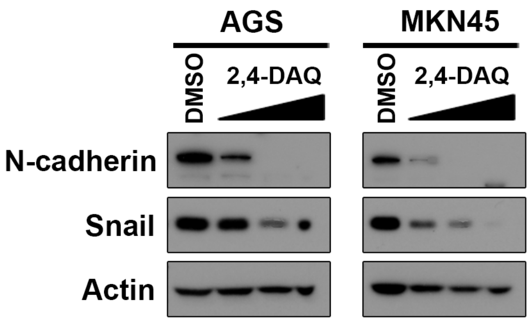

Figure S1. The expresion of N-cadherin and snail is downregulated by 2,4-DAQ

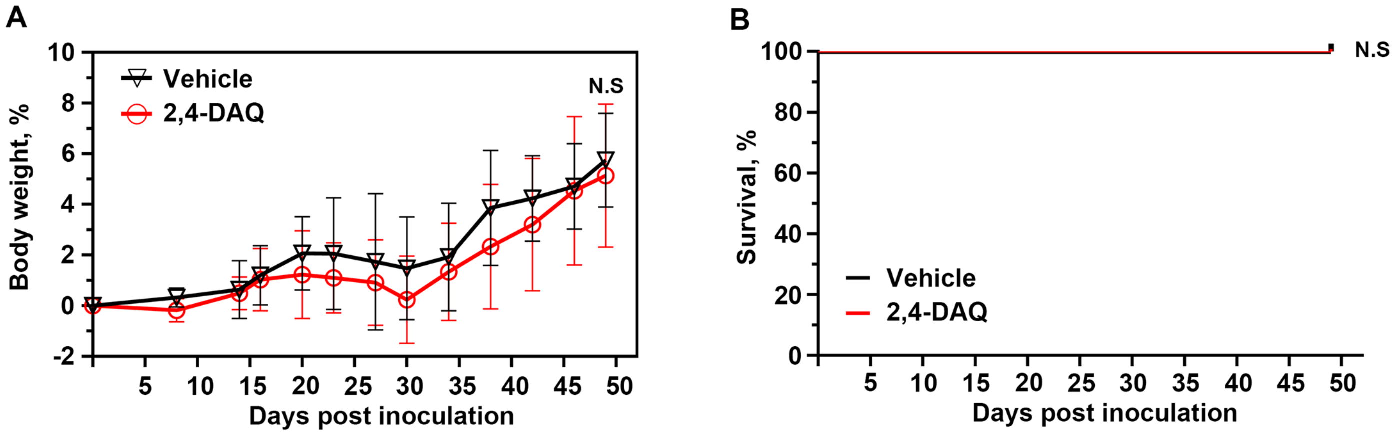

Figure S2. Effects of 2,4-DAQ on bodyweight change and survival rate in MKN45 xenograft nude mice.

(A) Bodyweight evaluation 2,4-DAQ vs. vehicle group. (B) Survival analysis by Log-rank test.

(N.S, not significant)

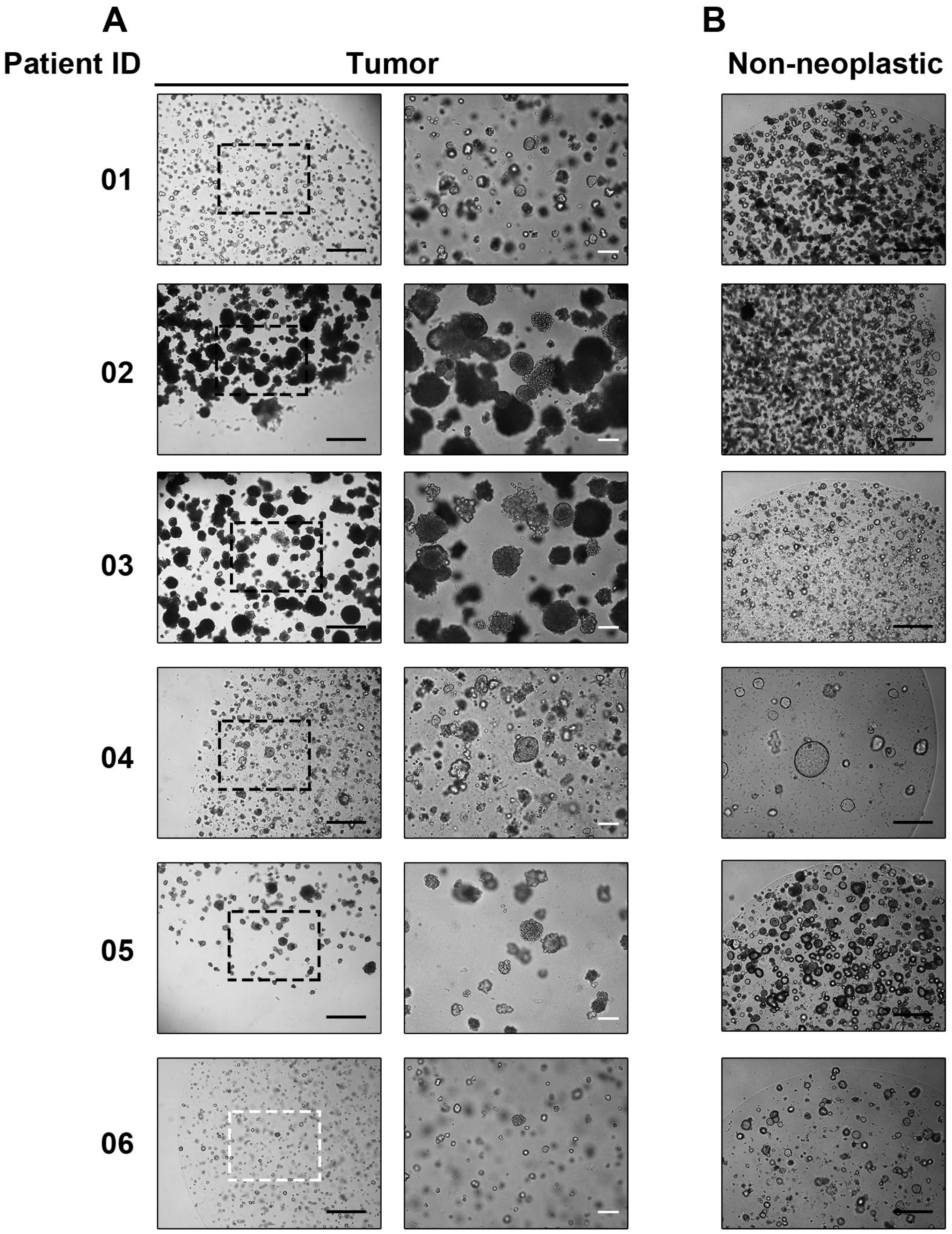

Figure S3. Establishment of PDOs from GC. Representative bright-field images of six (A) tumor-derived organoids and (B) non-neoplastic lesions-derived organoids. Black scale bars: 500 μm, white scale bars: 100 μm.

Supplementary Tables

Table S1. Summary of test compounds against Wnt signaling pathway

Compound	Target	Reference
2,4-diamino-quinazoline	β-catenin and TCF-dependent transcription	Z Chen et al. (2009) [15]
iCRT3	β-catenin interaction with TCF7L2	Bilir, et al. (2013) [S1]
iCRT14	β-catenin interaction with TCF7L2	Bilir, et al. (2013) [S1]
CCT031374 hydrobromide	β-catenin and TCF-dependent transcription	Ewan, et al. (2010) [S2]
PKF118-310	β-catenin and TCF-dependent transcription	Lepourcelet, et al. (2004) [S3]
PKF115-584	β-catenin interaction with TCF7L2	Doghman, et al. (2008) [S4]
Adavivint	Wnt pathway	Yazici, et al. (2017) [S5]
FH535	β-catenin and TCF-dependent transcription	Handeli and Simon (2008) [S6]
Quercetin (Flavonoids)	β-catenin and TCF-dependent transcription	Novo, et al. (2015) [S7]
Curcumin **(**Polyphenols**)**	Wnt pathway	He, et al. (2014) [S8]

S1 Bilir, B.; Kucuk, O.; Moreno, C. S., Wnt signaling blockage inhibits cell proliferation and migration, and induces apoptosis in triple-negative breast cancer cells. *J Transl Med* **2013,** 11, 280.

S2 Ewan, K.; Pajak, B.; Stubbs, M.; Todd, H.; Barbeau, O.; Quevedo, C.; Botfield, H.; Young, R.; Ruddle, R.; Samuel, L.; Battersby, A.; Raynaud, F.; Allen, N.; Wilson, S.; Latinkic, B.; Workman, P.; McDonald, E.; Blagg, J.; Aherne, W.; Dale, T., A useful approach to identify novel small-molecule inhibitors of Wnt-dependent transcription. *Cancer Res* **2010,** 70, (14), 5963-73.

S3 Lepourcelet, M.; Chen, Y.-N. P.; France, D. S.; Wang, H.; Crews, P.; Petersen, F.; Bruseo, C.; Wood, A. W.; Shivdasani, R. A., Small-molecule antagonists of the oncogenic Tcf/β-catenin protein complex. *Cancer Cell* **2004,** 5, (1), 91-102.

S4 Doghman, M.; Cazareth, J.; Lalli, E., The T cell factor/beta-catenin antagonist PKF115-584 inhibits proliferation of adrenocortical carcinoma cells. *J Clin Endocrinol Metab* **2008,** 93, (8), 3222-5.

S5 Yazici, Y.; McAlindon, T. E.; Fleischmann, R.; Gibofsky, A.; Lane, N. E.; Kivitz, A. J.; Skrepnik, N.; Armas, E.; Swearingen, C. J.; DiFrancesco, A.; Tambiah, J. R. S.; Hood, J.; Hochberg, M. C., A novel Wnt pathway inhibitor, SM04690, for the treatment of moderate to severe osteoarthritis of the knee: results of a 24-week, randomized, controlled, phase 1 study. *Osteoarthritis Cartilage* **2017,** 25, (10), 1598-1606.

S6 Handeli, S.; Simon, J. A., A small-molecule inhibitor of Tcf/beta-catenin signaling down-regulates PPARgamma and PPARdelta activities. *Mol Cancer Ther* **2008,** 7, (3), 521-9.

S7 Novo, M. C.; Osugui, L.; dos Reis, V. O.; Longo-Maugeri, I. M.; Mariano, M.; Popi, A. F., Blockage of Wnt/beta-catenin signaling by quercetin reduces survival and proliferation of B-1 cells in vitro. *Immunobiology* **2015,** 220, (1), 60-7.

S8 He, M.; Li, Y.; Zhang, L.; Li, L.; Shen, Y.; Lin, L.; Zheng, W.; Chen, L.; Bian, X.; Ng, H. K.; Tang, L., Curcumin suppresses cell proliferation through inhibition of the Wnt/beta-catenin signaling pathway in medulloblastoma. *Oncol Rep* **2014,** 32, (1), 173-80.

Table S2. Clinical information for GC PDOs

Patient ID	Age	Gender	Histologic Type (Lauren)	Histologic Grade	Pathologic Stage	
					pTNM	AJCC 8th
01	89	♀	Intestinal type	Moderately differentiated	pT2N1M0	IIA
02	69	♀	Diffuse type	Poorly differentiated	pT1aN0M0	IA
03	57	♀	Diffuse type	Poorly differentiated	pT1bN0M0	IA
04	67	♀	Diffuse type	Poorly differentiated	pT3N1M0	IIB
05	84	♂	Intestinal type	Moderately differentiated	pT3N2M0	IIIA
06	61	♂	Mixed type	Poorly differentiated	pT4aN3aM0	IIIB
